# Occurrence of Antibiotic Resistance in the Mediterranean Sea

**DOI:** 10.3390/antibiotics11030332

**Published:** 2022-03-03

**Authors:** Delia Gambino, Dario Savoca, Arianna Sucato, Valeria Gargano, Antonino Gentile, Licia Pantano, Domenico Vicari, Rosa Alduina

**Affiliations:** 1 Istituto Zooprofilattico Sperimentale della Sicilia “A. Mirri”, 90129 Palermo, Italy; deliagamb@gmail.com (D.G.); antogentile1980@gmail.com (A.G.); licia.pantano@izssicilia.it (L.P.); domenico.vicari@izssicilia.it (D.V.); 2 Department of Biological, Chemical and Pharmaceutical Sciences and Technologies (STEBICEF), University of Palermo, 90028 Palermo, Italy; dario.savoca@unipa.it (D.S.); sucato.arianna@gmail.com (A.S.)

**Keywords:** antibiotic-resistant bacteria, antibiotic resistance genes, *int1*, *bla*_TEM_, *sul*II, heavy metal resistance genes

## Abstract

Seawater could be considered a reservoir of antibiotic-resistant bacteria and antibiotic resistance genes. In this communication, we evaluated the presence of bacterial strains in seawater collected from different coasts of Sicily by combining microbiological and molecular methods. Specifically, we isolated viable bacteria that were tested for their antibiotic resistance profile and detected both antibiotic and heavy metal resistance genes. Both antibiotic-resistant Gram-negative bacteria, *Vibrio* and *Aeromonas*, and specific antibiotic resistance genes were found in the seawater samples. Alarming levels of resistance were determined towards cefazolin, streptomycin, amoxicillin/clavulanic acid, ceftriaxone, and sulfamethoxazole/trimethoprim, and mainly genes conferring resistance to β-lactamic and sulfonamide antibiotics were detected. This survey, on the one hand, presents a picture of the actual situation, showing the pollution status of the Tyrrhenian coast of Sicily, and, on the other hand, can be considered as a baseline to be used as a reference time for future analysis.

## 1. Introduction

Antibiotic resistance is one of the biggest public health challenges in every country over the world [1,2]. The economic costs, due to clinical interventions and productivity losses, account for 9 billion EUR and 20 billion USD in Europe and the USA, respectively. The spread of antibiotic-resistant bacteria (ARB) in the environment is linked to the overuse and misuse of antibiotics, and their uncontrolled release into waste in many parts of the world. Together with ARB, it is currently quite common to find antibiotic resistance genes (ARGs) in the environment, as well as in those that have never experienced antibiotic treatment [3]. The presence of ARGs in soil and environmental bacteria represents a threat to human health, while horizontal gene transfer mechanisms contribute to the diffusion of resistance determinants in pathogenic bacteria [4]. Hospitals, farms, aquacultures, and wastewater treatment plants (WWTPs) are considered “hotspot environments” where bacteria are exposed to high and repeated doses of antibiotics, have high growth rates because of nutrient abundance, and can be selected before being released into the natural environment [1]. Thus, antimicrobial agents and pathogenic-resistant bacteria can access sewage through the waste released from these “hotspots”, reaching water ecosystems with the final effluent [5]. ARGs are frequently associated with gene cassettes containing the class 1 integron, a potentially mobile genetic element responsible for the conjugative-mediated gene transfer, and heavy metal resistance genes (HMRGs) [6,7,8]. Thus, the concomitant presence of heavy metal and antibiotic resistance genes may favour the selection of multi-resistant bacteria and the spread of resistance into the environment [7]. Several studies reported the presence of antibiotic-resistant bacteria and/or resistance genes in different aquatic environments, such as surface waters [9,10], wastewater [11], and recreational coastal waters [12], and also in environments and animals presumably unaffected by anthropogenic factors, such as glaciers [13] and marine animals [6,14,15]. The aquatic environment can, therefore, be considered a reservoir of antibiotic resistance and the seawater as an important element that contributes to the transfer of resistance genes between bacterial species, related or not [16]. In particular, in coastal waters influenced by intense human activities and the presence of effluents and wastewater treatment plants, multi-resistant bacteria and ARGs could be essential indicators of contamination originating from the anthropogenic environment [17].

Hence, this study aims to evaluate the antibiotic resistance profile of bacteria isolated from seawater from different coastal locations in Sicily, by combining microbiological tests and molecular analysis for the detection of antibiotic and heavy metal resistance genes. This study has the scope, on the one hand, to inform us of the diffusion of AMR and ARGs in the seawater of the Tyrrhenian coast of Sicily, and, on the other hand, to present a picture of the current situation that could be compared in the future.

## 2. Results

### 2.1. Bacterial Isolation

In this study, a total of 29 bacterial strains were isolated from six seawater samples and subsequently identified by biochemical–enzymatic tests or PCR. Microbiological investigations led to the isolation of five different genera of bacteria with a higher percentage of Gram-negative (91%) among all the isolates (Table 1).

The most isolated bacterial strains belonged to *Vibrio* (44.8%) and *Aeromonas* (31%) genera; *Vibrio* spp. were isolated from all samples, while *Aeromonas* spp. were only isolated from the water samples of S. Flavia and Levanzo. Furthermore, *Aeromonas* spp. were the predominant genus in the S. Flavia samples, representing 70% of the isolates from the water samples from this site. Three other genera, *Bacillus*, *Klebsiella* and *Enterobacter*, were found, respectively, in the water coast samples of Lipari (40%), Acqua dei Corsari (25%), and Levanzo (25%); meanwhile, *E. coli* was found in the water coast samples of S. Flavia (10%) and Casteldaccia (67%). Finally, *Salmonella* spp. were not isolated from any of the samples tested.

### 2.2. Antibiotic Resistance Profile

**Figure 1:**
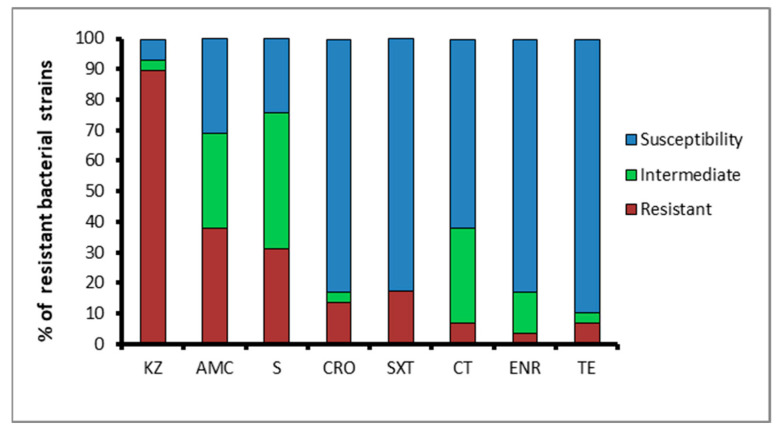
Percentage of isolates resistant, intermediate, or sensitive to antimicrobial agents: KZ, cefazolin; S, streptomycin; AMC, amoxicillin/clavulanic acid; CT, colistin; CRO, ceftriaxone; SXT, sulfamethoxazole/trimethoprim; TE, tetracycline; and ENR, enrofloxacin.

The antimicrobial resistance assay showed that almost all of the microbial isolates (with the exception of two susceptible isolates, one from Acqua dei Corsari and one from Levanzo) were resistant to cefazolin (89.6%), while 37.9% of the isolates displayed resistance to amoxicillin/clavulanic acid and 31% to streptomycin (Figure 1).

On the contrary, lower rates of antibiotic resistance to sulfamethoxazole/trimethoprim (17.2%), ceftriaxone (13.7%), colistin and tetracycline (both 6.8%), and enrofloxacin (3.4%) were exhibited. Surprisingly, high percentages of bacteria showing an intermediate resistance to streptomycin (44.8%), amoxicillin/clavulanic acid, and colistin (both 31%) were found (Figure 1). Moreover, the majority of the isolated bacteria (N = 11) did not show multiple resistance, displaying resistance to only one antibiotic. Nevertheless, a good number of the isolates (N = 7) were resistant to two of the eight antibiotics tested, and a small number of them were resistant to three (N = 3), four (N = 4) and five (N = 2) antibiotics. Notably, no one bacterial strain was resistant to six or seven antibiotics.

### 2.3. Antibiotic and Heavy Metal Resistance Genes

Metagenomic DNA was extracted from all the samples and analyzed by PCR for *bla*_TEM_, *bla_CTXM_*, *qnrS*, *sul*II, and *tet*(A) genes, which are the most frequent antibiotic resistance genes (Table 2).

All samples were positive for the presence of the *bla*_TEM_ gene, while no *bla_CTXM_* gene was detected. Only a few samples (*n* = 3), derived from the water coast samples of Lipari, Rometta, and Casteldaccia, were positive for the sulfonamide resistance gene (*sul*II). No *tet*(A) and *qnrS* genes were detected, according to the lower number of resistant bacteria isolated. In all water samples, no trace of the investigated antibiotics was detected (data not shown).

Moreover, the presence of the mobile element *int1*, as well as *czcA* and *arsB* heavy metal genes, was investigated. The *int1* gene, which encodes the class 1 mobile element integron, was found in all samples, whereas more than half of the samples (66.6%) were positive for *czcA* (cadmium, cobalt, and zinc resistance) and none were positive for *arsB* (arsenic resistance) genes (Table 2).

## 3. Discussion

In this study, we report the isolation and resistance profile of bacteria isolated from seawater from different locations on the Tyrrhenian coast of Sicily, Italy. The global consumption of antibiotics is directly reflected in their presence in various compartments of the environment, including the aquatic environment. As a result, aquatic environments, and especially coastal waters, are recognized as one of the reservoirs and pathways for the spread of antibiotic resistance. In Italy, several studies have been conducted on the presence of resistant bacteria in marine animals [6,14,18,19], but few have done the same for marine waters [6,10,20,21].

Our results confirm that Gram-negative bacteria, and in particular the genera *Aeromonas* and *Vibrio*, are found more frequently in samples from the marine environment. This finding is in accordance with other reports on marine samples, when both culture-dependent and metagenomic approaches were used [6,10,14,22]. The presence of these two genera in the coastal water of southern Italy was already assessed by other studies [6,21,23]. In fact, their physiological characteristics make them a part of the autochthonous microflora of marine waters, with consequent contaminations of sediments and aquatic organisms, such as seafood and sea turtles [15,21]. The incidence of these two genera in our samples is in agreement with what was previously detected by Pace et al. (2019) who report, in oral swabs of sea turtles in the Tyrrhenian coast of southern Italy, a higher incidence of *Vibrio* spp. (53.4%) in comparison with *Aeromonas* spp (17%) [15]. In contrast, two more recent studies conducted in Sicily reported a higher incidence of *Aeromonas* spp. in comparison with *Vibrio* spp. in Sicilian coastal environments. In fact, a study conducted on loggerhead sea turtle nesting samples recorded only *Aeromonas* spp. (55.6%) [24], while our group reported a prevalence of *Aeromonas* spp. (45.4%) compared with *Vibrio* spp. (9%) on the coastal water and sea turtles [6]. The abundance of isolates of *Aeromonas* spp. (70%) in the site of S. Flavia could be due to input of terrestrial origin, as the site is close to the estuary of a stream, an environment where the presence of these species is high [25]. In addition, the absence of these in the other samples could be due to water characteristics, such as salinity level, pH, and temperature, which could influence the distribution of both *Vibrio* spp. and *Aeromonas* spp., as demonstrated by previous studies [26,27]. However, the absence of information on these water parameters and the low number of samples analyzed does not explain the different distribution of *Aeromonas* spp. among the various sites. Finally, the presence of *Escherichia coli*, natural members of the intestinal microbiota of humans and animals, in the samples collected from S. Flavia and Casteldaccia could be an indication of faecal contamination due to the presence of pipelines near these two sampling sites [28,29].

Bacterial isolates were resistant to cefazolin (89.6%), amoxicillin/clavulanic acid (37.9%), and streptomycin (31%) according to the resistance levels (95.5%, 34.1%, and 43.2%, respectively) reported in a previous study conducted on wild marine species and water samples recovered in Sicily [6]. High resistance to cefazolin and streptomycin were also reported in marine waters from the southern coast of Turkey [30]. Moreover, lower rates of antibiotic resistance to sulfamethoxazole/trimethoprim (17.2%), tetracycline (6.8%), and enrofloxacin (3.4%) were exhibited. The low resistance to sulfamethoxazole/trimethoprim is an interesting result if we consider both the widespread use of this antibiotic and, above all, that another recent study conducted on marine species in Sicily recorded higher resistance values (37%) for this antibiotic [14], strongly suggesting that sea animals can act as concentrators and carriers of antibiotic-resistant bacteria [6,15]. The low resistance percentages of tetracycline and enrofloxacin were in accordance with those of the study mentioned above [6]. Only one strain showed resistance to enrofloxacin, data with positive implications for the marine environment if we consider that this antibiotic is generally used for therapies in veterinary centres; thus, a possible spreading of resistance mechanisms against enrofloxacin could affect the local fauna. Meanwhile, the low percentage of tetracycline resistance may be due to the photosensitive nature of this antibacterial, and its photochemical degradation is likely due to the warm and light-exposed conditions of the water [31], presumably decreasing the selective pressure of this antibiotic on the marine bacteria. The assessment of tetracycline levels in Sicilian waters could be used to clarify this aspect. These small numbers of resistant isolates could be reasonably linked to the lower bacterial density of seawater [6,32]. However, the presence of multi-resistant strains in coastal water samples highlights the dramatic implications for the health of marine organisms and human health [6]; some works attested to the presence of antibiotic-resistant strains in surfers and bathing waters, suggesting the dramatic consequences of the accidental ingestion of contaminated seawater [33]. Despite the limitations on the sample size of the present study, we surmise that collecting samples from more sites could confirm these results. Further studies using a bigger sample size and collecting samples over an extended period across various seasons and water dynamics would be of use in the future.

Classical cultivation, associated with biochemical and molecular identification, is of paramount importance in bacteriology because it allows the further analysis of the isolated bacterial strains as antibiotic resistance profiles. However, it is hampered by the difficulties in cultivating different bacteria with different growth needs and kinetic rates. Using metagenomic analysis of the 16S rDNA, we detected more than 100 genera in some of the samples analyzed here [10]. Thus, to have a deeper overview of antibiotic resistance, we analyzed the metagenomic samples to evaluate the presence of ARGs in the water. All samples were positive for the presence of the *bla*_TEM_ gene responsible for β-lactam resistance, while no *bla_CTXM_* gene was detected. β-lactam resistance was frequently observed in the marine ecosystem, particularly conferred by the presence of the *bla*_TEM_ gene [6]. Resistance to β-lactam antibiotics was frequently found in seawater and also in fishes and wild marine species such as sea turtles, which could be involved in the spread of this resistance [6,28,34]. The absence of the *bla_CTXM_* gene in Sicilian seawaters was in line with a previous study [6], while the absence of *tet*(A) and *sul*II genes is surprising if compared with the findings of some works that attest to their prevalent presence in surface water around the world, in Australia, Germany, and China, [9,35] and in fish farms and coastal environments [36].

Interestingly, the *int1* gene, encoding the mobile element class 1 integron, was found in all the samples. Previous studies hypothesized a positive correlation between the abundance of the class I integrons and the resistance against some metals (such asCd, Zn, and Pb) in freshwater sediment samples and bacterial isolates from water and shrimps [7,37,38]. Furthermore, the frequency of class I integrons has been postulated as an indicator of anthropogenic pollution in the environment [39]. In fact, the widespread presence of the gene *int1* in our samples highlights the potential transfer of ARGs and HMRGs between different bacterial strains and their migration between connected aquatic systems. Their diffusion into marine environments would increase the risk to human health because of the ineffectiveness of antibiotics for treating infectious bacterial diseases [40].

## 4. Materials and Methods

### 4.1. Sample Collection

**Figure 2:**
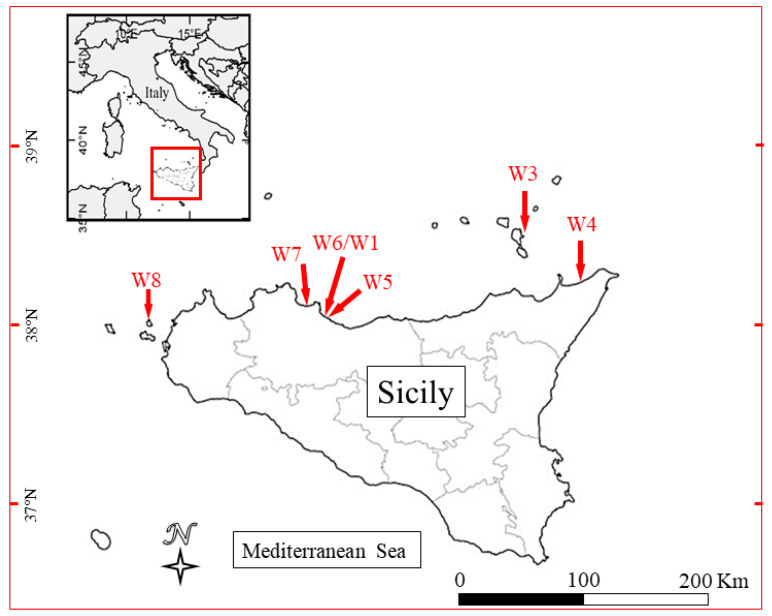
Map of the sampling site: Santa Flavia (W1/W6); Lipari (W3); Rometta (W4); Casteldaccia (W5); Acqua dei corsairi (W7); and Levanzo (W8).

Bacteria were isolated from the seawater analyzed during routine activities at the Istituto Zooprofilattico Sperimentale della Sicilia. Sampling was conducted during May–July 2019 in 6 different seaside locations on the Tyrrhenian coast of Sicily. Seawater samples (1 L) were collected at 5 m from the shore in Lipari, Casteldaccia, S. Flavia, Rometta, Levanzo, and Acqua dei Corsari (Figure 2). Sampling points were chosen close to residential areas and farms.

Samples were transferred to sterile biological bottles for laboratory analyses, and kept at 4 °C until their processing. All the samples investigated in this study are reported in Table 1.

### 4.2. Bacterial Isolation

For bacterial isolation, the water was filtered with membranes with pores of 0.45 µm (±0.02 µm) diameter. The membranes were then placed in tubes containing 10 mL of alkaline peptone water and incubated at 37 °C for 24–48 h. Each sample was then seeded onto Columbia agar with 5% sheep blood and selective media, namely: (i) Mannitol salt agar, (ii) MacConkey agar, and (iii) thiosulfate-citrate-bile salts-sucrose agar. In addition, to isolate the *Salmonella* spp. possibly present, before seeding in xylose lysine desoxycholate agar and brilliant green agar, the second enrichment in the selenite cystine and Rappaport-Vassiliadis broth was made, incubating both broths and plates at 37 °C for 24–48 h. All of the media were purchased from Oxoid. The isolated strains were identified by biochemical–enzymatic tests, such as catalase, oxidase, mobility, indole, sugar fermentation, citrate, and urea metabolism, as previously described [24]. When the biochemical analysis was not exhaustive, amplification and sequencing of the 464 bp fragment of the 16S rDNA were carried out. An aliquot (2 µL) of the bacterial lysate, prepared as previously described [41], was used to amplify the 464 bp internal fragment of the 16S rDNA using One Taq DNA polymerase (NEB), using primer pairs and the corresponding annealing temperatures. After confirmation through agarose (1% *w*/*v*) gel electrophoresis, the polymerase chain reaction (PCR) products were purified and sequenced at Macrogen Inc. (Seoul, Korea, sequencer). The nucleotide sequences were identified using the NCBI nucleotide BLAST.

### 4.3. Antibiotic Susceptibility Test

The antibiotic susceptibility of the isolated bacterial strains was performed using the Kirby–Bauer method on Mueller–Hinton agar, by testing their sensitivity to eight antibiotics, namely, amoxicillin/clavulanic acid (AMC, 30 µg), cefazolin (KZ, 30 µg), ceftriaxone (CRO, 30 µg), colistin (CT, 10 µg), streptomycin (S, 10 µg), enrofloxacin (ENR, 5 µg), sulfamethoxazole/trimethoprim (SXT, 25 µg), and tetracycline (TE, 30 µg), as described elsewhere [42]. Interpretation of the results was carried out by referring to the Clinical and Laboratory Standards Institute (CLSI) range [43]. Antimicrobial disks were obtained from Oxoid (Hampshire, United Kingdom). The selected set included antibiotics commonly used in human and veterinary medicine; most of them were classified by the World Health Organization as important antimicrobials in human medicine and included in the Critically Important Antimicrobials (CIA) list [44].

### 4.4. Detection of Antibiotic and Heavy Metal Resistance Genes

The metagenomic DNA was extracted from 6 water samples using the protocol reported in [45]. Metagenomic DNA was utilized as a template to amplify the genes coding for products responsible for the resistance to antimicrobials, such as tetracycline *tet*(A), sulfonamides *sul*II, β-lactams, *bla*_TEM_ and *bla_CTXM_*, and quinolones *qnrS*. Moreover, the *int1*, *arsB,* and *czcA* genes were investigated. As a control, the 142 bp DNA fragment of the 16S rDNA gene was used. All PCR reactions were performed using the annealing temperature and the primer pairs listed in [6]. The presence of the expected amplification product was considered as a positive sample.

## 5. Conclusions

The presence of antibiotic resistance in marine waters is now well documented and attributable to the excessive use of antibiotics in human and animal fields; these usually reach the sea through wastewater, or simply from a river. As a consequence, marine waters can become a possible source of antibiotic resistance not only for wild marine organisms and species bred in mariculture plants (usually molluscs, sea bass, sea bream, or salmon), but also for humans. We can acquire antibiotic resistance through the consumption of edible marine species, such as fish and molluscs, or through direct contact with seawater. Our data show that water samples collected in Sicily in coastal sites influenced by human activities, bathing areas, and the proximity of residential centres, contain antibiotic resistance genes and that these are more present in the most polluted sites. The Gram-negative bacteria *Vibrio* and *Aeromonas* were found in the samples. Strains resistant towards cefazolin, streptomycin, amoxicillin/clavulanic acid, ceftriaxone, and sulfamethoxazole/trimethoprim, and mainly genes conferring resistance to β-lactmic and sulfonamide were detected.

The findings of this study could be a starting point for further investigations, to evaluate the spread of antibiotic-resistant bacteria in the marine environment, their dispersal mechanisms, and the potential factors involved in horizontal gene transfer phenomena in the aquatic medium.

## Figures and Tables

**Table 1:** Details of the location of the sampling site and the relative bacterial isolates.

Location	Coordinate	Isolated Bacterial Genera	Number of Isolates
Lipari W3	38°29′13.3″ N 14°57′59.2″ E	*Vibrio* *Bacillus*	3
			2
Casteldaccia W5	38°03′21.3″ N 13°32′38.9″ E	*Vibrio*	1
		*Escherichia coli*	2
S. Flavia W6 + W1	38°03′53.3″ N 13°32′17.2″ E + 38°03′52.7″ N 13°32′16.5″ E	*Vibrio*	2
		*Aeromonas*	7
		*Escherichia coli*	1
Acqua dei Corsari W7	38°05′54.8″ N 13°24′49.6″ E	*Vibrio*	3
		*Klebsiella*	1
Levanzo W8	37°59′33.1″ N 12°21′01.6″ E	*Vibrio*	1
		*Aeromonas*	2
		*Enterobacter*	1
Rometta W4	38°14′16.5″ N 15°24′50.0″ E	*Vibrio*	3

**Table 2:** Summary of the presence/absence of ARGs, HMRGs, and *int1* gene in the 6 metagenomic samples analyzed.

Location	*bla*_TEM_	*qnrS*	*sul*II	*tet*(A)	*bla_CTXM_*	*czcA*	*arsB*	*int1*
Lipari W3	+	ND ^1^	+	ND	ND	+	ND	+
Casteldaccia W5	+	ND	+	ND	ND	ND	ND	+
S. Flavia W6	+	ND	ND	ND	ND	+	ND	*+*
Acqua dei corsari W7	+	ND	ND	ND	ND	ND	ND	+
Levanzo W8	+	ND	ND	ND	ND	+	ND	+
Rometta W4	+	ND	+	ND	ND	+	ND	+

^1^ ND indicates that the correct amplification product was not obtained.
